# The anticancer effects of MPT0G211, a novel HDAC6 inhibitor, combined with chemotherapeutic agents in human acute leukemia cells

**DOI:** 10.1186/s13148-018-0595-8

**Published:** 2018-12-29

**Authors:** Huang-Ju Tu, Yi-Jyun Lin, Min-Wu Chao, Ting-Yi Sung, Yi-Wen Wu, Yi-Ying Chen, Mei-Hsiang Lin, Jing-Ping Liou, Shiow-Lin Pan, Chia-Ron Yang

**Affiliations:** 1 School of Pharmacy, College of Medicine, National Taiwan University, No.33, Linsen S. Road, Taipei, 10050 Taiwan; 2 Graduate Institute of Cancer Molecular Biology and Drug Discovery, College of Medical Science and Technology, Taipei Medical University, Taipei, Taiwan; 3 Ph.D Program in Biotechnology Research and Development, College of Pharmacy, Taipei Medical University, Taipei, Taiwan; 4 Ph.D Program for Cancer Molecular Biology and Drug Discovery, College of Medical Science and Technology, Taipei Medical University and Academia Sinica, Taipei, Taiwan; 5 School of Pharmacy, College of Pharmacy, Taipei Medical University, Taipei, Taiwan; 6 Biomedical Commercialization Center, Taipei Medical University, Taipei, Taiwan

**Keywords:** Histone deacetylase 6, Acute leukemia, Combination therapy, Ku70, Microtubule dynamics

## Abstract

**Background:**

There are some limitations of standard chemotherapy for acute leukemia. Vincristine and doxorubicin are commonly used for acute leukemia, but they may induce serious side effects such as cardiomyopathy and neurotoxicity. Furthermore, chemotherapy resistance occurs more and more frequently. Therefore, effective treatment strategies are needed. Histone deacetylase 6 inhibition is considered as a potential therapeutic strategy for acute leukemia, since it is observed that HDAC6 is overexpressed in acute leukemia and regulates tumor survival. Combination therapy for cancer is used to minimize adverse drug effects, reduce drug dosage, enhance efficacy, and prevent drug resistance. In order to improve efficacy of chemotherapy agents of acute leukemia, this study will investigate the effects of combination MPT0G211, a novel histone deacetylase 6 inhibitor, with doxorubicin or vincristine on human acute leukemia cells.

**Results:**

MPT0G211 combined with doxorubicin induces DNA damage response on human acute myeloid leukemia cells. MPT0G211 can additionally increase Ku70 acetylation and release BAX to mitochondria. Ectopic expression of HDAC6 successively reversed the apoptosis triggered by the combined treatment. Moreover, co-treatment of MPT0G211 and vincristine may alter microtubule dynamics, triggering acute lymphoblastic leukemia cells arrest in mitotic phase followed by induction of the apoptotic pathway. Finally, MPT0G211 plus doxorubicin or vincristine can significantly improve the tumor growth delay in a tumor xenograft model.

**Conclusions:**

Collectively, our data highlighted that MPT0G211 in combination with chemotherapy drugs has significant anticancer activity, suggesting a novel strategy for the treatment of acute leukemia.

**Electronic supplementary material:**

The online version of this article (10.1186/s13148-018-0595-8) contains supplementary material, which is available to authorized users.

## Background

Leukemia is a hematologic malignancy caused by the rapid proliferation of abnormal blood cells. This disease may be acute or chronic. In the former type, most cancer cells remain in a more immature and frequently dividing “blast” form, which leads to a range of complications and is fatal without proper treatment [1].

Acute leukemia can be further divided in two subtypes by lineage. Of these, acute lymphocytic leukemia (ALL), which is characterized by the abnormal proliferation of lymphocytes (e.g., B cells and T cells), is the most common type of pediatric cancer. Acute myeloid leukemia (AML) involves the abnormal proliferation of immature myeloid progenitors (e.g., granulocytes, monocytes, red blood cells, and platelets). Both subtypes can progress quickly but differ considerably in terms of survival (5 year survival: 67.5% and 25.9% for ALL and AML, respectively) [2].

Currently, the chemotherapeutic agents vincristine (VCR) and doxorubicin (DOXO) are often used to treat acute leukemia. However, the clinical use of these drugs is often limited by the risk of side effects such as cardiomyopathy [3] and neurotoxicity [4]. Accordingly, safer, effective treatment strategies for acute leukemia are needed.

Histone deacetylases (HDACs) enzymatically regulate DNA expression by removing acetyl groups from histones. As a result, DNA can more tightly wind around histones, thereby decreasing gene expression [5]. Mammals harbor four classes of HDACs, which can be distinguished by structure, enzymatic function, subcellular localization, and expression pattern [6]. Notably, HDAC overexpression has been identified in many types of cancer, indicating that HDACs’ activity may be a promising therapeutic target for cancer management [7]. To date, the US Food and Drug Administration has approved four pan-HDAC inhibitors for the treatment of various cancers, especially hematological malignancies such as cutaneous T cell lymphoma [8], peripheral T cell lymphoma [9], and multiple myeloma [10]. Although pan-HDAC inhibitors differ in terms of chemical structure, they are similarly cardiotoxic, and therefore, their clinical utility is limited [11]. Emerging evidence shows a potential association of pan-HDAC inhibitor-induced cardiotoxicity with specific HDAC isoforms, leading to an interest in isoform selectivity in the field of drug development [12].

The HDAC family member HDAC6 resides mainly in the cytoplasm, where it plays a unique role in regulating non-histone proteins without affecting gene expression [13]. Many types of cancers express high levels of HDAC6 [14–16], and this overexpression can be exploited for treatment purposes. For example, the synergistic effects of HDAC6 inhibition and bortezomib induced apoptotic cell death in ovarian cancer cells [17], whereas HDAC6 knockdown markedly reduced the migration and invasion activity of hepatocellular carcinoma cells [18]. Another recent study identified HDAC6 as an important regulator of the cytoskeleton, which is a required component for invasive activity in breast cancer cells [19].

In the present study, we evaluated the effects of a novel, selective HDAC6 inhibitor, MPT0G211, in acute leukemia cells when administered alone or in combination with chemotherapy drugs. We found that in AML cells, MPT0G211 potentiated the cytotoxic effects of DOXO by impairing DNA repair machinery and activating Bcl-2-associated X protein (BCL-XL)-dependent cell apoptosis. Additionally, when combined with VCR, MPT0G211 disrupted microtubule dynamics to induce mitotic arrest in ALL cells. These data suggest that HDAC6 inhibition represents a novel opportunity in the treatment of acute leukemia.

## Materials and methods

### Cell lines

The human acute myeloid leukemia cell line, HL-60, human acute lymphoblastic leukemia cell line, MOLT-4, and human umbilical vein endothelial cells (HUVEC) were obtained from Bioresource Collection and Research Center (Taiwan). Cells were maintained in RPMI-1640 medium supplemented with 10% (*v*/*v*) fetal bovine serum (Gibco, Carlsbad, CA, USA) and 1% of a mixture of penicillin-streptomycin-amphotericin B (Kibbutz Beit Haemek, Israel). For HDAC6 overexpressed cells, HL-60 cells were transfected with HDAC6-FLAG (Plasmid #13823, Addgene Inc., Cambridge, MA, USA) by using Turbofect (Thermo Fisher Scientific, Rockford, IL, USA). All cells were cultured at 37 °C in a humidified atmosphere with 5% CO2.

### Chemicals and antibodies

MPT0G211, tubastatin A (TBA), and SAHA were synthesized by Dr. Jing-Ping Liou’s Lab. (School of Pharmacy, College of Pharmacy, Taipei Medical University, Taiwan), and the purity are more than 98%. Doxorubicin (DOXO), cyclophosphamide (CTX), and vincristine (VCR) was obtained from Cayman Chemical (Ann Arbor, MI, USA). Antibodies against BCL-2, BCL-XL, cleaved caspase 3, caspase 8, caspase 9, acetyl-α-tubulin, acetyl-histone 3, histone 3, HDAC6, survivin, p-ATM, p-ATR, p-CHK1, CHK1, cyclin B1, aurora B, p-PLK1, p-H3S10, p-CDC2 (Y15), and p-CDC2 (T161) were purchased from Cell signaling (Danvers, MA, USA). α-Tubulin, γ-H2AX, ATM, ATR, BAX, cytochrome c, and COX IV were from Genetex (Irvine, CA, USA). PARP and CDC2 were from Santa Cruz Biotechnology (Dallas, TX, USA).

### Cell viability assay

Cells were seeded in a 24-well plate at a density of 1 × 10^6^ cells/well and then treated with various concentrations of MPT0G211, TBA, DOXO, VCR or CTX alone or in combination treatment for 48 h. MTT solution (final concentration 0.5 mg/ml) was then added to the 24-well plate in the dark, and the plate was incubated at 37 °C for 2 h. For HL-60 cells, 10% SDS were added in the wells to dissolved the crystal, and for HUVEC cells, MTT-containing medium were removed and DMSO were added to each well to lyse cells; the absorbance was spectrophotometrically analyzed at 570 nm.

### Protein extraction and Western blot

Cells were treated with indicated condition and then lysed in lysis buffer (10 mmol/L Tris-HCl (pH 7.4), 150 mmol/L NaCl, 1 mmol/L EGTA, 1 mmol/L PMSF, 10 μg/mL aprotinin, 10 μg/mL leupeptin, 1 mM sodium orthovandate, 1 mM NaF, and 1% Triton X-100) for 30 min and centrifuged at 13,000 rpm. The supernatants were quantified by BCA Protein Assay Kit (Thermo Fisher Scientific, Rockford, IL, USA). Equal amounts of protein were separated by SDS-PAGE and then transferred to PVDF membranes. The membranes were blotted with different antibodies overnight at 4 °C and conjugated with appropriate secondary antibodies.

### Immunoprecipitation

After treated with MPT0G211, TBA, or DOXO, cells were lysed in lysis buffer containing Halt™ protease and phosphatase inhibitor (Thermo Fisher Scientific, Rockford, IL, USA). Meanwhile, Protein A Magnetic Beads (Bio-Rad, Hercules, CA, USA) were incubated with capture antibodies and rotated 10 min at room temperature. The mixture was rinsed with PBST three times to remove unbounded antibodies and incubated with cell lysate for 1 h. The complexes captured by magnetic rack were then washed three times using PBST to remove unbound proteins. All immunoprecipitation samples were suspended in Laemmli sample buffer and boiled for 10 min. The complex proteins were then analyzed by Western blotting.

### Flow cytometry

After the treatment of vehicle (0.1% DMSO), MPT0G211, TBA, or chemotherapy agents for the indicated time courses, the cells were fixed with 75% (*v*/*v*) alcohol at 4 °C overnight. After centrifugation, cells were incubated in DNA extraction buffer (0.2 M NaHPO_4_–0.1 M citric acid) for 20 min at room temperature. Then, the cells were centrifuged and resuspended with 0.5 mL PI solution (1% Triton X-100, 100 μg/mL RNAase A and 80 μg/mL propidium iodide). Evolution of the cell cycle histogram was analyzed with the FACScan and CellQuest software (Becton Dickinson).

### Cellular dissection method

Nuclear/Cytosol Fractionation Kit (Biovision, Inc., Milpitas, CA, USA) was used to separate cytosol and nuclear. Briefly, cells were collected and centrifuged at 600*g* for 5 min, supernatants were removed, and lysate were resuspended in Cytosol Extraction Buffer-A, vortex vigorously for 15 s and placed on ice for 10 min. Cytosol Extraction Buffer-B were then added to the mixture, vortex for 5 s, incubated on ice for 1 min, and centrifuged at 14,500 rpm to acquire cytosolic fraction. The remaining pallets were resuspended in nuclear extraction buffer, vortex the sample for 15 s, and returned the sample to ice for 10 min. After repeated for four times, samples were centrifuged at 14,500 rpm to acquire nuclear extraction.

Cytochrome c Releasing Apoptosis Assay Kit (Biovision, Inc., Milpitas, CA, USA) was used to separate mitochondria and cytosol. Briefly, cells were centrifuged at 600*g* for 5 min, supernatant was removed, and cytosol extraction buffer was added for 10 min. Cells were homogenized in an ice-cold Dounce tissue grinder and transferred homogenate to a new tube. The mixture was centrifuged at 700*g* for 10 min, supernatant was collected into a fresh tube and centrifuged at 10,000*g* for 30 min to acquire cytosolic fraction. The pellet was resuspended in mitochondrial extraction buffer and vortex 10 s to obtain mitochondria fraction.

### Immunofluorescence

To observe microtubule distribution, cells were treated with MPT0G211, TBA alone, or in combination with vincristine for 24 h. The cells were fixed with 4% paraformaldehyde for 15 min then permeabilized with 0.1% Tritin X-100 for 10 min. After washing with PBST for several times, 4% BSA were used to block non-specific proteins for 1 h then washed with PBST again and incubated with primary antibody α-tubulin for 2 h. FITC-conjugated anti-mouse IgG antibody were then used for another 2 h. Finally, cover slides were recovered to the slides with mounting gel containing DAPI stain. Images were detected and captured with the ZEISS confocal microscope.

### Tumor xenograft model

Seven-week-old male severe combined immunodeficiency mice were fed ad libitum water and Pico-Lab Rodent Diet. All procedures were performed in accordance with the NIH guidelines on laboratory animal welfare approved by the Animal Use and Management Committee of Taipei Medical University (IACUC No. LAC-2015-0163). HL-60 or MOLT-4 cells (1 × 10^7^ cells in 0.2 ml PBS) were subcutaneously injected into the flanks of the mice. When tumor sizes reached 200 mm^3^, mice were randomized into four groups with an indicated dosage of DOXO, VCR, and MPT0G211 alone or in combination treatment. All mouse tumors were allowed to reach an endpoint volume of 1200 mm^3^.

### Statistical analysis

All data were expressed as mean values ± S.E.M. and were done independently three times. The significance of differences between the experimental groups and controls was assessed by Student’s *t* test. *P* < 0.05 was considered statistically significant (*p < 0.05; **p < 0.01; ***p < 0.001; compared with the respective control group).

## Results

### MPT0G211 induces apoptosis in acute leukemia cells

**Fig. 1:**
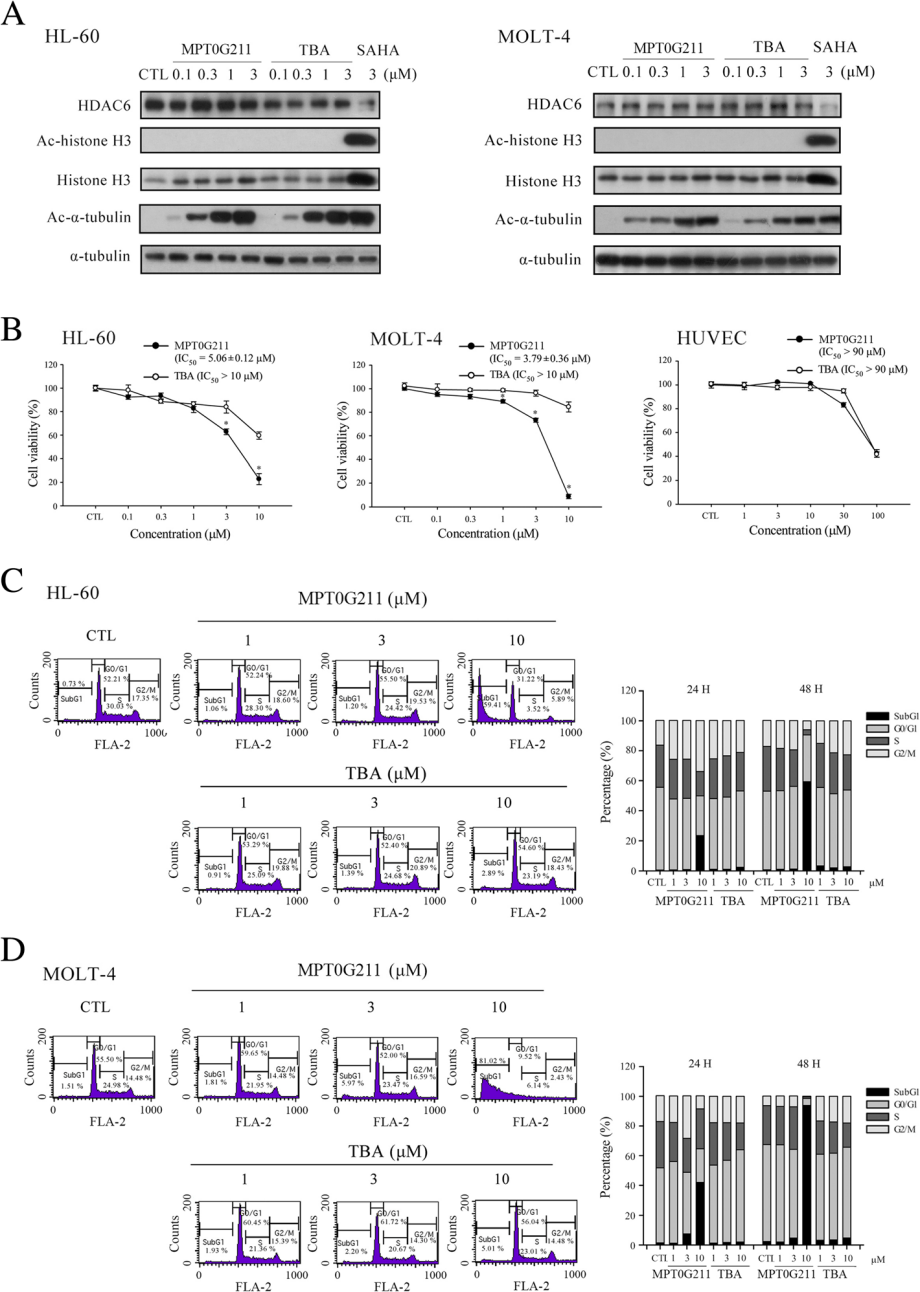
The effects of MPT0G211 on HL-60 and MOLT-4 cell growth and cell cycle progression. **a** Acetyl-α-tubulin and acetyl-histone 3 were detected in cells treated with MPT0G211 or tubastatin A (TBA) for 24 h. **b** HL-60, MOLT-4, and HUVECs were incubated with different concentrations of MPT0G211 or TBA for 48 h. Cell viability was evaluated using an MTT assay. **c** HL-60 and **d** MOLT-4 cells were treated with MPT0G211 or TBA for 48 h and analyzed by flow cytometry to assess cell cycle distribution. Data are shown as means ± standard errors of the means. *p < 0.05 versus the TBA-treated group

In our previous study, we showed that MPT0G211 is a selective HDAC6 inhibitor with more potent activity than the currently available HDAC6 inhibitor ACY-1215 [20]. In this study, we examined the inhibitory effects of MPT0G211 on HDAC6 activity in acute leukemia cells. As shown in Fig. 1a, MPT0G211 more strongly induced α-tubulin acetylation when compared with tubastatin A (TBA) without affecting histone 3 acetylation in both HL-60 human acute myeloid leukemia cells and MOLT-4 human acute lymphoblastic leukemia cells. Furthermore, MPT0G211 inhibited HDAC6 enzyme activity without significantly affecting the HDAC6 protein levels.

We additionally used an MTT assay to evaluate the effects of HDAC6 inhibitors on the viability of acute leukemia cells (Fig. 1b). Notably, MPT0G211 more effectively induced HL-60 and MOLT-4 cell death (IC_50_ = 5.06 ± 0.12 and 3.79 ± 0.36 μM, respectively), compared with TBA (IC_50_ > 10 μM for both cell types), but had no cytotoxic effect on normal human umbilical vein endothelial cells (HUVECs) (IC_50_ > 90 μM). Subsequently, we used a flow cytometric assay to investigate the effect of MPT0G211 on cell cycle distribution in cells that had been treated with MPT0G211 and TBA for 24 and 48 h. Compared with TBA, treatment with 10 and 3 μM MPT0G211 increased the proportions of HL-60 and MOLT-4 cells, respectively, and in the sub-G1 phase at both 24 and 48 h, the effect in the latter cell type was slight but statistically significant (Fig. 1c, d). These data suggest that MPT0G211 is a potent and selective HDAC6 inhibitor that specifically targets malignant cancer cells.

### MPT0G211 sensitizes HL-60 cells to doxorubicin-induced cell death

**Fig. 2:**
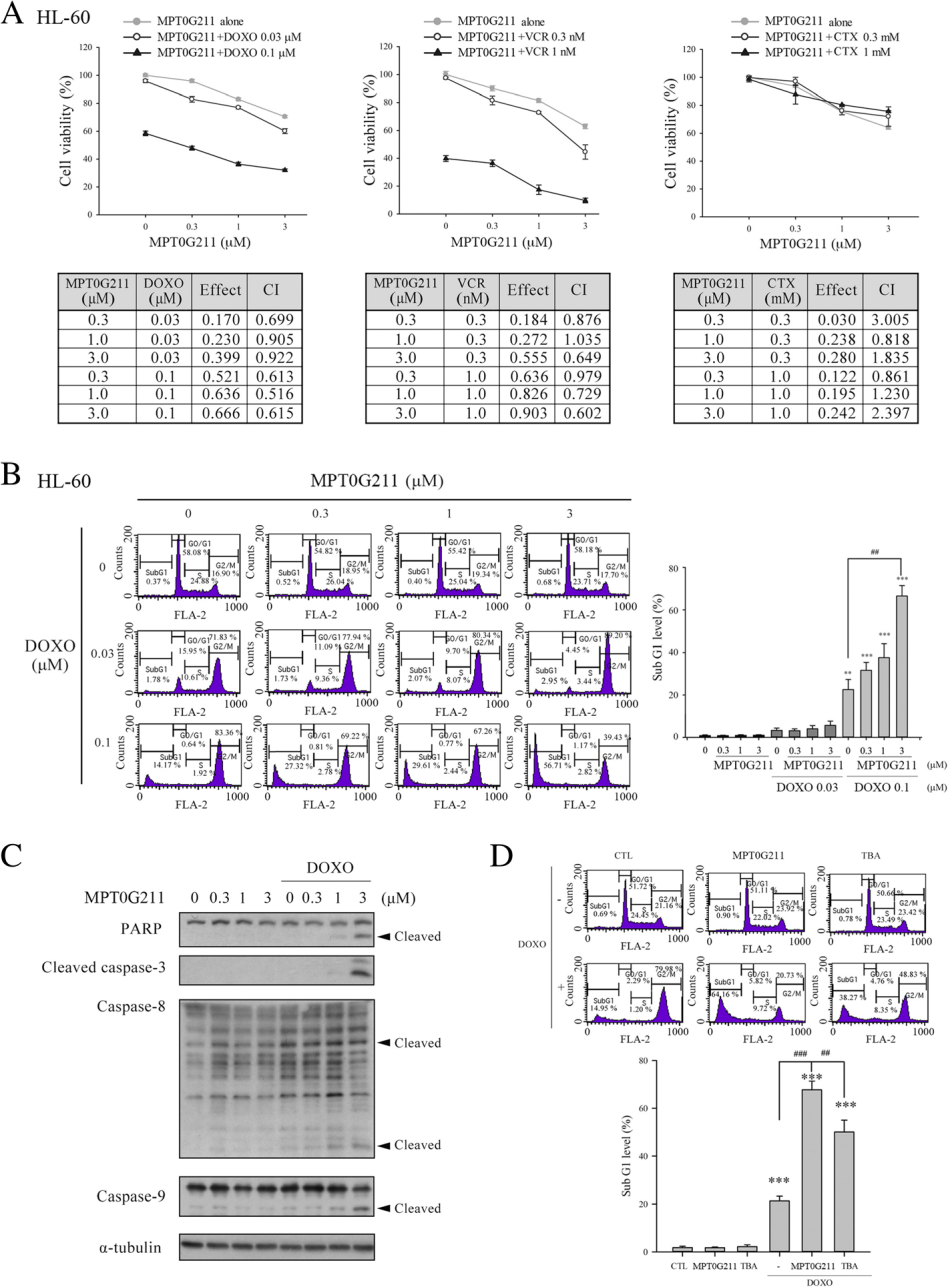
The combination of MPT0G211 and doxorubicin had a synergistic effect on HL-60 cell death. **a** The viability of cells treated with MPT0G211 and different doses of doxorubicin (DOXO), vincristine (VCR), and cyclophosphamide (CTX) for 48 h was tested. The synergistic effects of these drugs were evaluated using the combination index (CI), with values of > 1.0, 1.0, and < 1.0 indicating an antagonistic, additive, or synergistic interaction, respectively. **b** Cell cycle distribution was determined in cells treated with various concentrations of MPT0G211 and DOXO for 48 h. **c** The combined effect of MPT0G211 and DOXO on the expression of caspases 3, 8, and 9 and poly-ADP ribose polymerase (PARP). **d** Flow cytometry was used to evaluate the proportion of cells in the sub-G1 phase of cell cycle following incubation with 3 μM MPT0G211 or tubastatin A (TBA) combined with 0.1 μM DOXO. Data are shown as means ± standard errors of the means. *p < 0.05, **p < 0.01, and ***p < 0.001 versus the control group

Combination therapy regimens are usually used to increase the efficacies of chemotherapy drugs, prevent drug resistance, and reduce unwanted side effects during the treatment of acute leukemia. In this study, we subjected HL-60 cells to combined treatment regimens of MPT0G211 with three chemotherapy drugs currently used for acute leukemia: DOXO, VCR, and cyclophosphamide (CTX). The combination index (CI) was used to evaluate the effects of these two-drug combinations, with CI values of 1, > 1, and < 1 indicating addiction, antagonism, and synergism, respectively [21]. As shown in Fig. 2a, MPT0G211 (0.3, 1, or 3 μM) acted synergistically with DOXO and VCR against HL-60 cells, whereas no obvious synergistic effect was observed with CTX.

Anthracycline drugs such as DOXO are considered standard treatment options for AML. Therefore, we evaluated the effect of a combination of MPT0G211 and DOXO in HL-60 cells. Flow cytometry revealed that this combination significantly induced cell cycle arrest in the G2/M phase, with the subsequent accumulation of cells in the sub-G1 phase (Fig. 2b). Similar effect was also noted in the other AML cell line, MV4-11 (Additional file 1). Moreover, the levels of the apoptotic proteins caspases 3, 8, and 9 and poly-ADP ribose polymerase (PARP) were also increased in these cells following combination treatment (Fig. 2c). TBA was then used to confirm that the synergistic effects of MPT0G211 were mediated through HDAC6 inhibition. In that experiment, TBA also increased the proportion of sub-G1 phase cells when administered in combination with DOXO, although its effects were less potent than those of MPT0G211 (Fig. 2d).

### MPT0G211 acetylates Ku70 and regulates Ku70-Bax binding to impair the DNA repair machinery initiated by doxorubicin

**Fig. 3:**
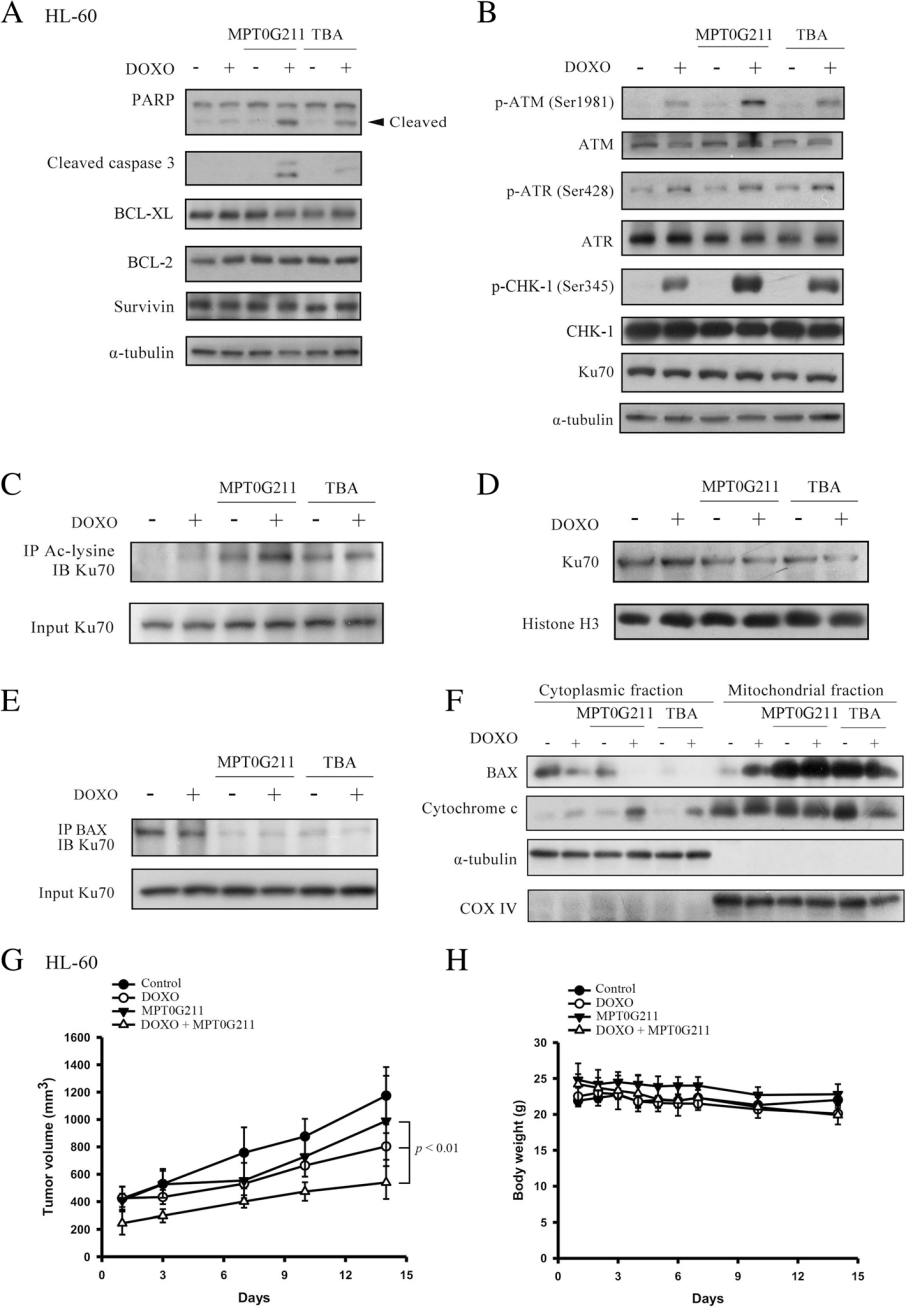
MPT0G211 potentiates doxorubicin-induced cytotoxicity by increasing apoptosis and decreasing DNA repair machinery in HL-60 cells. **a** The levels of the apoptotic proteins caspase 3 and poly-ADP ribose polymerase (PARP) and the pro-survival proteins BCL-XL, BCL-2, and survivin were determined in cells treated with MPT0G211 (3 μM) or tubastatin A (TBA) (3 μM) in combination with doxorubicin (DOXO) (0.1 μM) for 24 h. **b** Co-treatment of MPT0G211 or TBA with DOXO increased the phosphorylation of ATM, ATR, and CHK1 proteins. **c** The cells were incubated with MPT0G211 or TBA plus DOXO for 24 h, after which total cell lysates were immunoprecipitated with an anti-acetyl-lysine antibody and immunoblotted for Ku70. **d** Nuclear Ku70 protein levels were measured in cells treated with MPT0G211 or TBA in combination with DOXO. **e** Cells were incubated with MPT0G211 or TBA plus DOXO for 36 h, after which total cell lysates were immunoprecipitated with an anti-BAX antibody and immunoblotted for Ku70. **f** Cellular distributions of cytochrome c and BAX after co-treatment with MPT0G211 or TBA plus DOXO. **g** Antitumor activity of MPT0G211 plus doxorubicin in a HL-60 xenograft model. When the tumor size reached 200 mm^3^, mice were injected with vehicle, doxorubicin (1 mg/kg, i.p., q3d), and MPT0G211 (30 mg/kg, i.p., qd) alone or a combination of both. The curves of tumor growth volume were expressed as mean ± SEM. **h** Changes of body weight after treatment

To elucidate the mechanism by which combination therapy induces apoptotic cell death, we examined the levels of the pro-survival proteins BCL-2, BCL-XL, and survivin but observed no changes after treatment with MPT0G211, TBA, DOXO, or a combination treatment (Fig. 3a). Therefore, we considered the DNA damage response, a process which is centrally regulated by proteins such as ATM and ATR, which cause cell cycle arrest [22] and recruit DNA repair proteins such as Ku70 [23]. Notably, DOXO treatment induced significant phosphorylation of ATM, ATR, and CHK1, which was further enhanced by the combination of MPT0G211 and DOXO; in other words, MPT0G211 can potentiate the DOXO-induced DNA damage response (Fig. 3b).

Furthermore, combined MPT0G211 and TBA treatment induced the acetylation of Ku70 (Fig. 3c), which remained sequestered in the cytosol; accordingly, Ku70 could not sufficiently bind to double-strand break sites to promote DNA repair (Fig. 3d) [24]. In a previous study, acetylation of Ku70 was found to disrupt its bonds with BAX; consequently, free BAX translocates into the mitochondria to induce apoptotic cell death [25]. In this study, we showed that MPT0G211 significantly reduced the binding of Ku70 to BAX (Fig. 3e). We used cell fractionation to further confirm that MPT0G211 facilitates the release of cytochrome c to the cytoplasm (Fig. 3f) to induce caspase-mediated cell apoptosis. To evaluate that the synergistic effect of DOXO and MPT0G211 can be clinically relevant, we performed this co-treatment in established HL-60 tumor xenograft. Once tumors were palpable (approximately 200 mm^3^), mice were randomized into control (vehicle) and treatment groups. As shown in Fig. 3g, combination treatment significantly potentiated the antitumor activity of DOXO. The mice tolerated all of the treatments without overt signs of toxicity (Fig. 3h). Taken together, these data indicated that co-treatment of DOXO and MPT0G211 markedly suppressed tumor growth both in vitro and in vivo.

### HDAC6 overexpression reverses the synergistic effects of MPT0G211 and doxorubicin

**Fig. 4:**
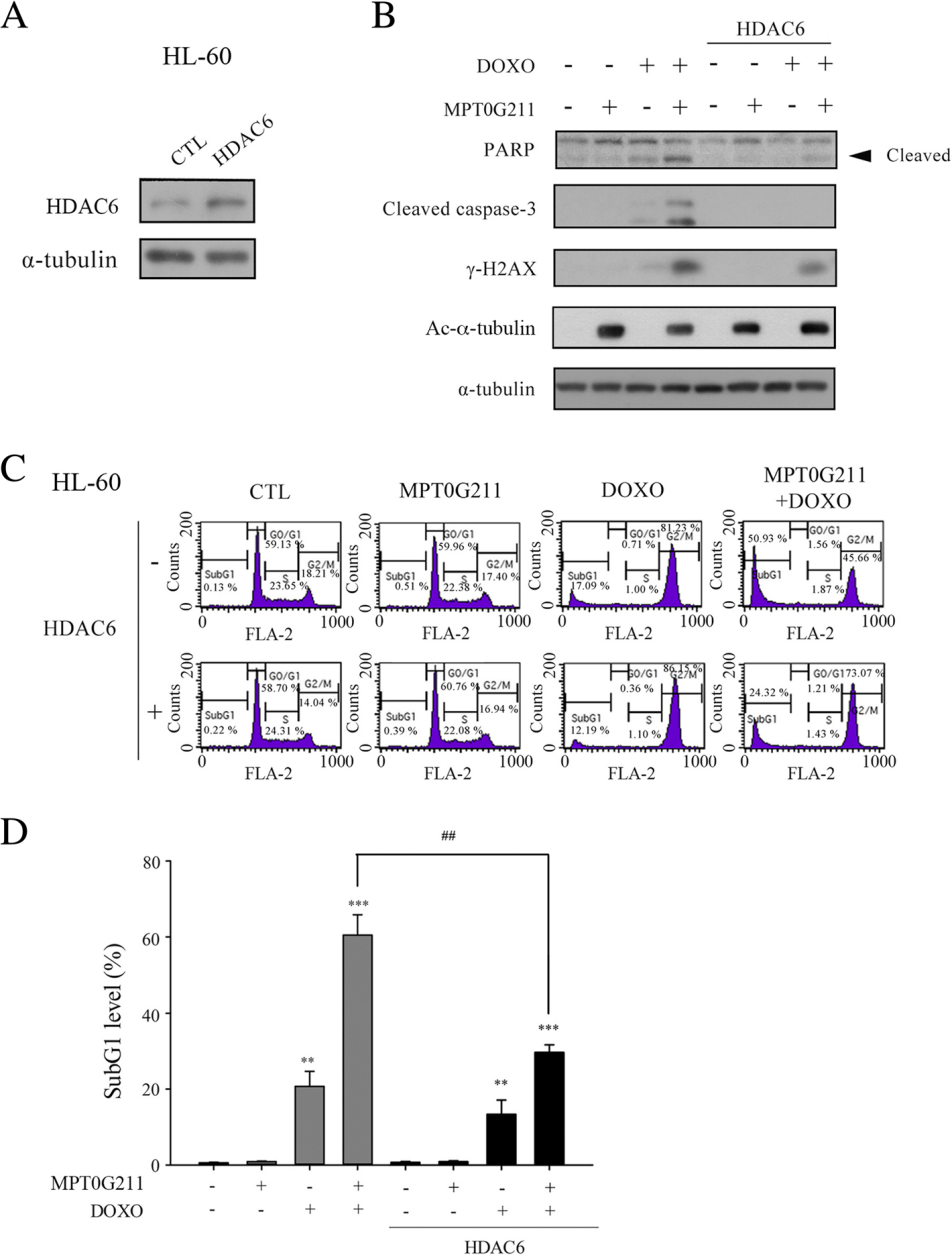
Ectopic expression of HDAC6 rescues cells from apoptosis induced by MPT0G211/doxorubicin combination. **a** HDAC6 levels were measured 18 h after transfection with an HDAC6-expression plasmid. **b** Following co-treatment with MPT0G211 and doxorubicin (DOXO) for 24 h, the levels of the apoptotic proteins poly-ADP ribose polymerase (PARP), caspase 3, γ-H2AX, and acetyl-α-tubulin were measured in HDAC6-overexpressing cells. **c** Cell cycle distribution was measured in HDAC6-transfected HL-60 cells following treatment with a combination of MPT0G211 and DOXO. A quantitative analysis of the proportions of cells in the sub-G1 phase of cell cycle is shown in **d**. Data are shown as means ± standard errors of the means. *p < 0.05, **p < 0.01, and ***p < 0.001 versus the control group

To validate that the synergistic effect of MPT0G211 and DOXO is truly mediated by the inhibition of HDAC6, we examined whether HDAC6 overexpression could rescue cells from apoptosis triggered by the combined treatment (Fig. 4a). Here, ectopic expression of HDAC6 effectively reduced the expression of caspase 3, PARP, and the DNA damage marker γ-H2AX (Fig. 4b) while decreasing the proportion of cells in the sub-G1 phase of cell cycle (Fig. 4c, d). Together, these data suggest that the synergistic effects of MPT0G211 and DOXO are mediated through HDAC6 inhibition.

### MPT0G211 potentiates vincristine-induced cell arrest on MOLT-4 cells

**Fig. 5:**
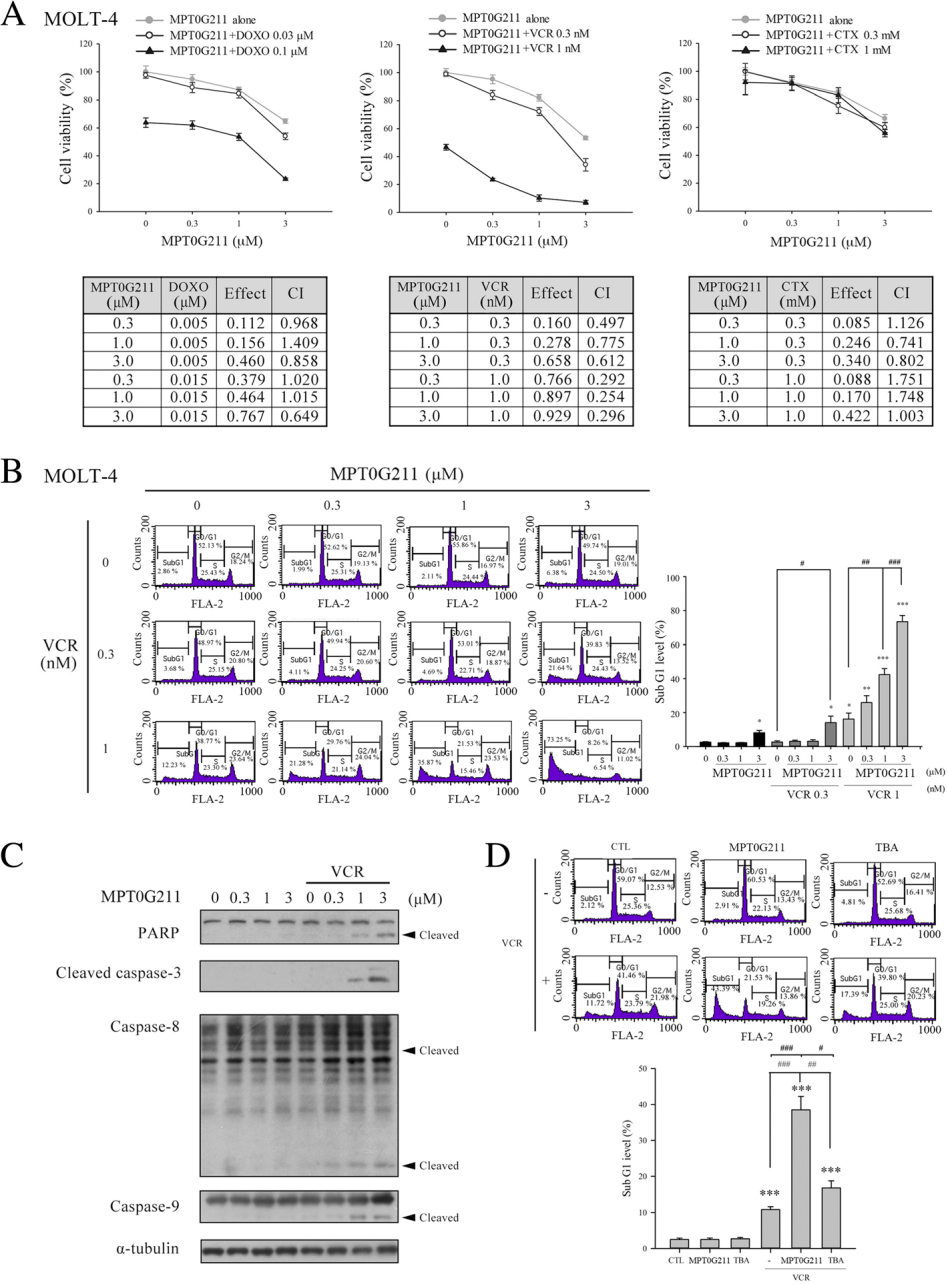
MPT0G211 potentiates vincristine-induced apoptosis in MOLT-4 cells. **a** The combined effects of MPT0G211 with doxorubicin (DOXO), vincristine (VCR), or cyclophosphamide (CTX) at the indicated concentrations were evaluated after a 48-h treatment. **b** Cell cycle distribution was determined after cells were treated with a combination of MPT0G211 and VCR for 48 h. **c** The combined effects of MPT0G211 and VCR on the expression of caspases 3, 8, and 9 and poly-ADP ribose polymerase (PARP). **d** Flow cytometry was used to evaluate the proportions of cells in the sub-G1 phase of cell cycle after incubation with 3 μM MPT0G211 or tubastatin A (TBA) together with 1 nM VCR for 48 h. Data are shown as means ± standard errors of the means. *p < 0.05, **p < 0.01, and ***p < 0.001 versus the control group

We also tested the combined effects of MPT0G211 plus DOXO, VCR, or CTX on MOLT-4 cells (ALL). As shown in Fig. 5a, MPT0G211 (0.3, 1, or 3 μM) and various concentrations of VCR exhibited synergistic effects on the viability of MOLT-4 cells. MPT0G211 induced an increase of cells in the sub-G1 phase at 48 h in a concentration-dependent manner (Fig. 5b) and induced cell apoptosis via caspase pathway activation and PARP cleavage when combined with VCR (Fig. 5c). And this synergistic effect can also be confirmed in other ALL cell line, CCRF-CEM (Additional file 1). Although TBA also increased the proportion of cells in the sub-G1 phase when used in combination with VCR, this drug had less potent effects than did MPT0G211 (Fig. 5d).

### Combined effects of MPT0G211 and vincristine on microtubule dynamics

**Fig. 6:**
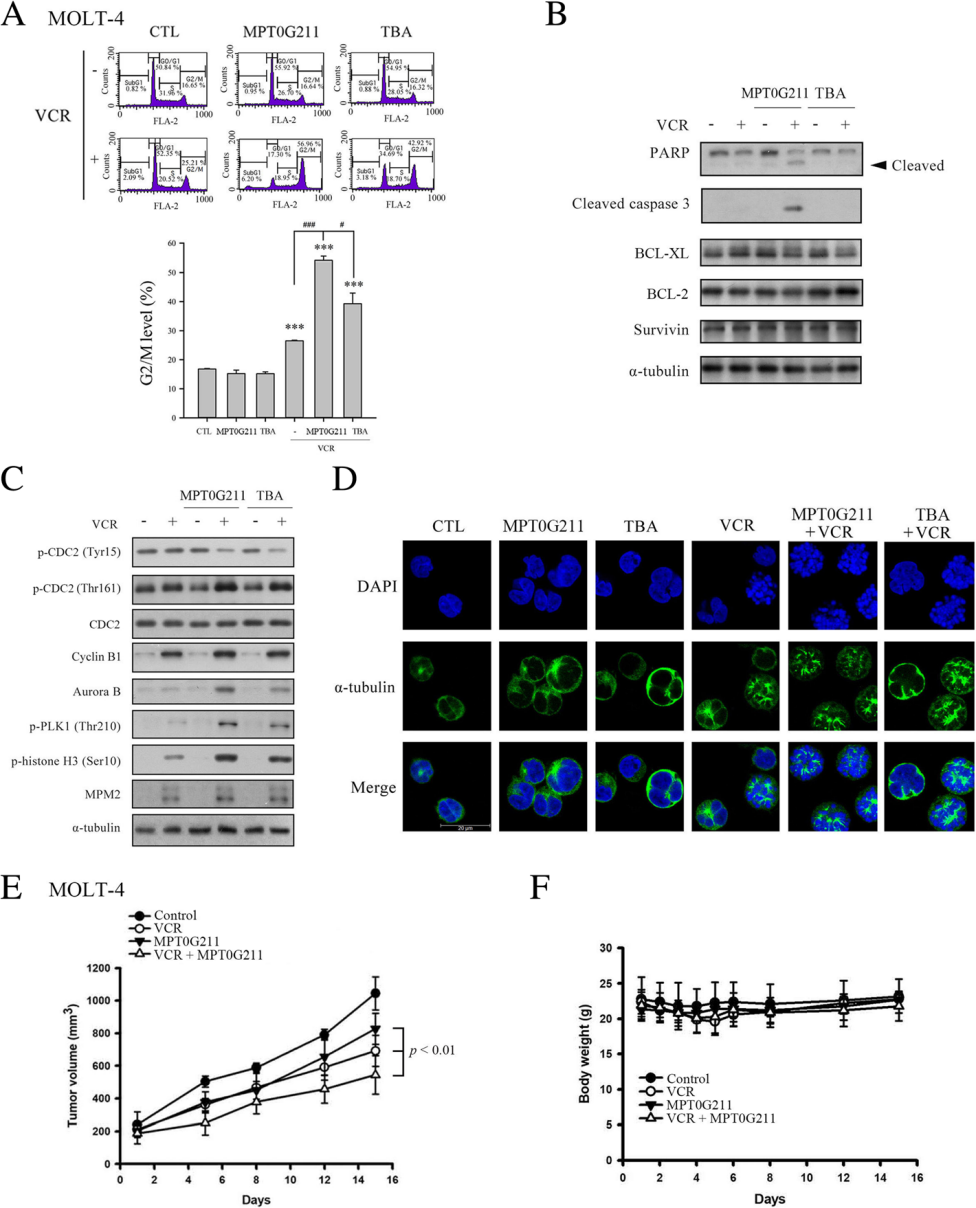
MPT0G211 sensitized MOLT-4 cells to vincristine-mediated mitotic arrest. **a** Cell cycle distributions of cells exposed to MPT0G211, tubastatin A (TBA), vincristine (VCR), or the indicated combination therapy for 24 h. A statistical analysis of the proportions of cells in the G2/M phase is shown in the right panel. **b** The levels of the apoptotic proteins caspase 3 and poly-ADP ribose polymerase (PARP) and the pro-survival proteins BCL-XL, BCL-2, and survivin were determined in cells treated with MPT0G211 (3 μM) or TBA (3 μM) in combination with VCR (1 nM) for 24 h. **c** The protein levels of cyclin B1, aurora B, p-CDC2, p-PLK, p-Histone 3, and MPM2 were evaluated following treatment with a combination of MPT0G211 or TBA with VCR for 24 h. **d** Cells were co-treated with MPT0G211 or TBA with VCR for 24 h and incubated with an α-tubulin antibody or DAPI. Microtubule dynamics were evaluated using a ZEISS LS 510META confocal microscope (magnification × 630). Scale bar = 20 μM. **e** Antitumor activity of MPT0G211 plus vincristine in a MOLT-4 xenograft model. When the tumor size reached 200 mm^3^, mice were injected with vehicle, vincristine (1 mg/kg, i.p., qwk), and MPT0G211 (30 mg/kg, i.p., qd) alone or a combination of both. The curves of tumor growth volume were expressed as mean ± SEM. **f** Changes of body weight after treatment. Data are shown as means ± standard errors of the means. *p < 0.05, **p < 0.01, and ***p < 0.001 versus the control group

VCR exerts its action by binding to tubulin to inhibit mitosis during metaphase and cause cell apoptosis [26]. We found that MPT0G211 and VCR synergistically led to an increase in cells in the G2/M phase after 24 h (Fig. 6a), although treatment with these drugs alone or in combination did not affect the levels of the pro-survival proteins BCL-2, BCL-XL, and survivin (Fig. 6b). However, the drug combination increased the expression of the M phase-regulating proteins MPM2, polo-like kinase (PLK), aurora B, and cyclin B1; additionally, it enhanced the phosphorylation of CDC2 at Thr161 but suppressed phosphorylation at Tyr15 (Fig. 6c). Finally, immunofluorescence images revealed that the combination of VCR and MPT0G211 more strongly altered microtubule polymerization, compared with VCR alone (Fig. 6d). We further evaluate whether MPT0G211 could enhance antitumor effect of VCR in mice bearing MOLT-4 xenograft. As shown in Fig. 6e, co-treatment of VCR and MPT0G211 exhibited significant antitumor activity in MOLT-4 tumor xenograft. No significant body weight difference or other adverse side effect was observed (Fig. 6f). Together, these results indicated that concomitant VCR and MPT0G211 can potentiate VCR- induced cell death both in vitro and in vivo.

## Discussion

Although pan-HDAC inhibitors have been approved for the treatment of cancers, currently, they can only be administered to subset of patients with selected hematological malignancies. The use of these drugs is further limited by the frequent clinical observation of negative side effects such as electrocardiographic changes, characterized by T-wave flattening, ST segment depression, and QT interval prolongation [11]. In contrast to other HDACs, HDAC6 regulates the acetylation of non-histone proteins such as α-tubulin [27], β-catenin [28], cortactin [29], and heat shock protein 90 [30] and participates in cancer development and progression [13]. The finding that HDAC6-deficient mice is effectively normal, unlike mice deficient in other HDAC isoforms, suggests that HDAC6 inhibition will cause few adverse effects [31]. Therefore, our finding that HDAC6 inhibition by MPT0G211 or TBA did not cause cytotoxic effects on HUVECs is consistent with previous reports. Specifically, we found that although α-tubulin acetylation was observed even at low doses (0.1 μM) of MPT0G211, high concentrations were required to observe the antitumor effects of this drug (IC_50_ = 5.06 and 3.79 μM in HL-60 and MOLT-4 cells, respectively; Fig. 1). Our findings and previous studies in which HDAC6 inhibitors were shown to act synergistically with chemotherapy drugs [32] suggest that such combination therapies may be a rational strategy for the use of HDAC6 inhibitors in cancer treatment.

DOXO, a topoisomerase II inhibitor that induces DNA breakage via intercalation, was reported to induce an increase in the proportion of HL-60 cells in the G2/M phase of cell cycle [33]. Consistent with that report, we observed that although MPT0G211 itself had no effect on cell cycle distribution at concentrations below the IC_50_ value, it potentiated DOXO-induced G2/M arrest (Fig. 2b). This phenomenon might be due to activation of the ATR-CHK1 pathway [34], as additional research has shown that HDAC6 depletion increases cisplatin-induced cytotoxicity by activating the ATR/CHK1 pathway in non-small cell lung cancer [35]. Hence, we suggest that MPT0G211 enhances the ability of DOXO to induce ATM, ATR, and CHK1 activation and, consequently, G2/M arrest (Fig. 3b).

The DNA repair process is an important mediator of resistance to DNA targeting drugs [36]. Ku70, a DNA repair protein that binds to the double-strand break ends caused by DOXO, has been reported as a target of HDAC6 [37]. In our study, MPT0G211 induced Ku70 acetylation. This led to the sequestration of Ku70 in the cytosol, which blocked its binding to double-strand breaks (Fig. 3c, d) and therefore impaired DOXO-induced DNA repair. Furthermore, acetyl-Ku70 promoted the dissociation of Ku-70 from BAX, thus promoting BAX-dependent cell apoptosis (Fig. 3e, f). Together, these findings demonstrate the multifaceted ability of MPT0G211 to potentiate the cytotoxic effects of DOXO. We further proved that this ability of MPT0G211 is dependent on the inhibition of HDAC6, as the observed synergistic effects could be reversed by the ectopic expression of HDAC6 protein (Fig. 4).

Currently, microtubule-destabilizing agents such as VCR are also considered important for the treatment of ALL. At high concentrations, VCR binds to low-affinity binding sites on microtubule to induce disorganization, whereas at lower concentrations, it interferes with microtubule dynamics without altering polymer levels, leading to mitotic spindle disruption [38]. A previous study demonstrated an increased frequency of multipolar spindle formation in cells co-treated with paclitaxel and the HDAC6 inhibitor, ACY-241 [39]; in other words, the combination of an HDAC6 inhibitor and VCR may also yield therapeutic benefits. Indeed, we found that MPT0G211 potentiated the mitotic arrest induced by VCR (Fig. 5). We further observed that co-treatment with these two drugs altered the phosphorylation pattern of CDC2, which binds to cyclin B1 and promotes the G2/M transition [40]. Specifically, this combination suppressed the inhibitory phosphorylation of Tyr15 on CDC2 while enhancing the phosphorylation of Thr161 and cyclin B1, which suggests a synergistic effect of these drugs on M phase arrest.

During mitosis, the proteins aurora B and Polo-like kinase 1 are involved in centrosome maturation and mitotic spindle formation [41]. In this study, we demonstrated that co-treatment with MPT0G211 and VCR activated these proteins. Using immunofluorescence images, we also demonstrated that treatment with MPT0G211 or TBA alone had no effect on microtubule distribution, whereas combined treatment with MPT0G211 and VCR led to significant increases in abnormal long astral microtubule formation and chromosome distribution (Fig. 6c). These results were consistent with a previous study in which a combination of the non-selective HDAC inhibitor vorinostat and VCR induced changes in microtubule dynamics [42]. Previously, the HDAC6 inhibitor tubacin was shown to slow microtubule growth by promoting the interaction of HDAC6 with tubulin in a tubulin acetylation-independent manner [43]. Therefore, MPT0G211 and VCR likely disrupt microtubule dynamics via different mechanisms but act synergistically in combination. We also provide compelling evidence that combined treatment of VCR with MPT0G211 synergistically inhibits the growth of human acute lymphoblastic leukemia cells in animal xenograft.

## Conclusion

**Fig. 7:**
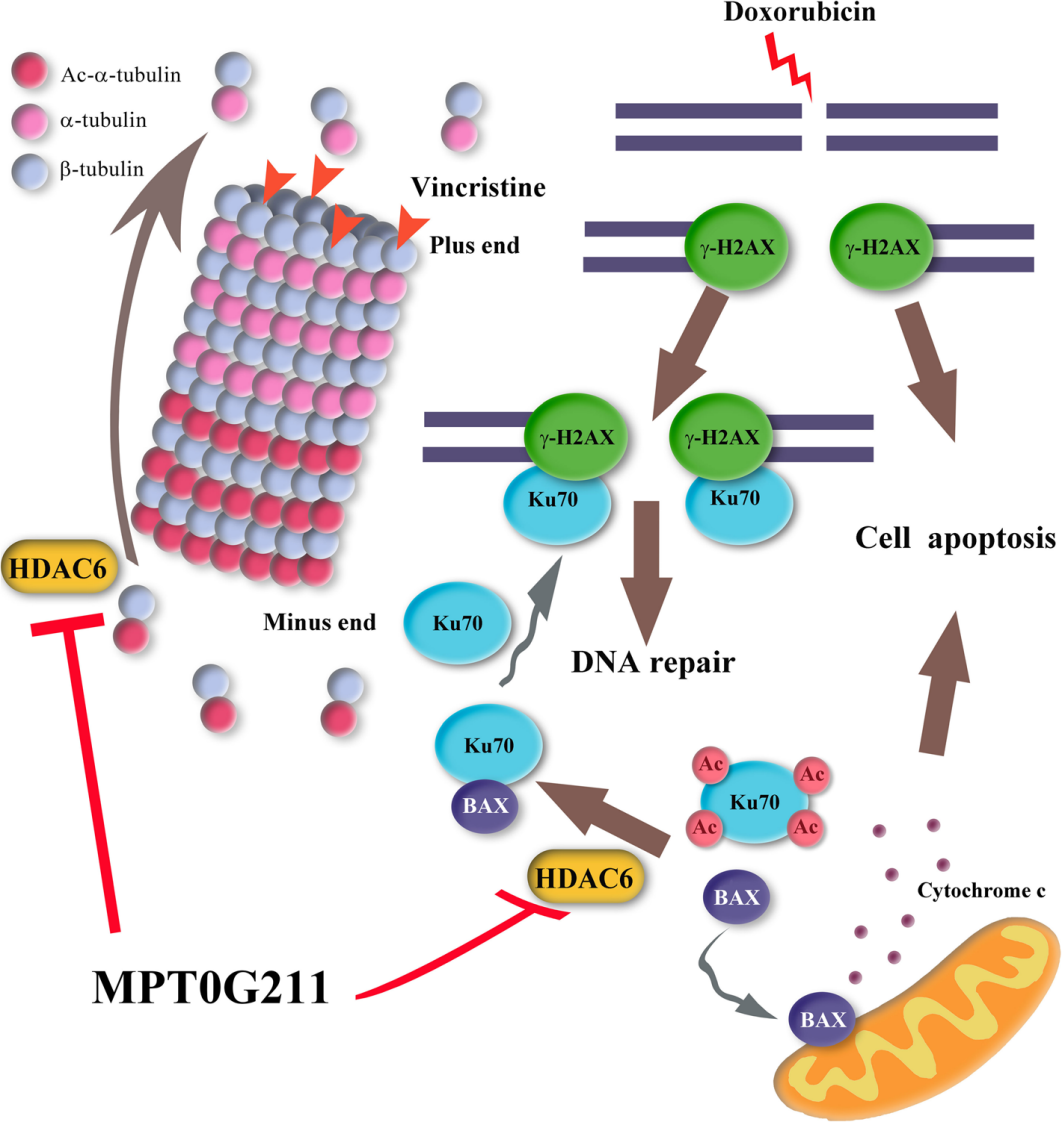
Schematic mechanisms mediated by MPT0G211 in combination with doxorubicin or vincristine in acute leukemia cell lines. When combined with doxorubicin (DOXO), MPT0G211 induces Ku70 acetylation and impairs DNA repair machinery. Moreover, acetylated Ku70 releases BAX, thus promoting BAX-mediated apoptotic cell death. MPT0G211 also synergizes with vincristine (VCR) to disrupt microtubule dynamics

In conclusion, our data suggest that compared with TBA, MPT0G211 is a more potent and selective HDAC6 inhibitor than current TBA. Furthermore, the combination of MPT0G211 and DOXO markedly inhibited HL-60 cell survival via apoptosis and interference with DNA repair machinery. Moreover, the combination of MPT0G211 and VCR significantly disrupted microtubule dynamics and induced M stage arrest in MOLT-4 cells (Fig. 7). As HDAC6 inhibitors are at an early stage of clinical development, our findings may provide a novel therapeutic strategy and future applications for cancer therapy.

## Additional file

Additional file 1: **Figure S1.** The combination effect of MPT0G211 and chemotherapeutic agent on acute leukemia cell lines. (A) The viability of cells treated with MPT0G211 and different doses of doxorubicin (DOXO) or vincristine (VCR) for 48 h was tested on CCRF-CEM and MV4-11 cell lines. The synergistic effects of these drugs were evaluated using the combination index (CI), with values of > 1.0, 1.0, and < 1.0 indicating an antagonistic, additive, or synergistic interaction, respectively. (B) Cell cycle distribution was determined in cells treated with various concentrations of MPT0G211 and DOXO for 48 h on MV4-11 cells. (DOCX 197 kb)

## Abbreviations

ALL: Acute lymphocytic leukemia
AML: Acute myeloid leukemia
CTX: Cyclophosphamide
DOXO: Doxorubicin
HDAC: Histone deacetylase
HUVEC: Human umbilical vein endothelial cells
TBA: Tubastatin A
VCR: Vincristine
