# Supplementary material for: The anticancer effects of MPT0G211, a novel HDAC6 inhibitor, combined with chemotherapeutic agents in human acute leukemia cells
Source: Clin Epigenetics. 2018 Dec 29;10:162. doi: 10.1186/s13148-018-0595-8 (PMC6310984; doi:10.1186/s13148-018-0595-8)
Supplement: Supplementary file 1 — Figure S1. The combination effect of MPT0G211 and chemotherapeutic agent on acute leukemia cell lines. (A) The viability of cells treated with MPT0G211 and different doses of doxorubicin (DOXO) or vincristine (VCR) for 48 h was tested on CCRF-CEM and MV4-11 cell lines. The synergistic effects of these drugs were evaluated using the combination index (CI), with values of > 1.0, 1.0, and < 1.0 indicating an antagonistic, additive, or synergistic interaction, respectively. (B) Cell cycle distribution was determined in cells treated with various concentrations of MPT0G211 and DOXO for 48 h on MV4-11 cells. (DOCX 197 kb) [file 13148_2018_595_MOESM1_ESM.docx]

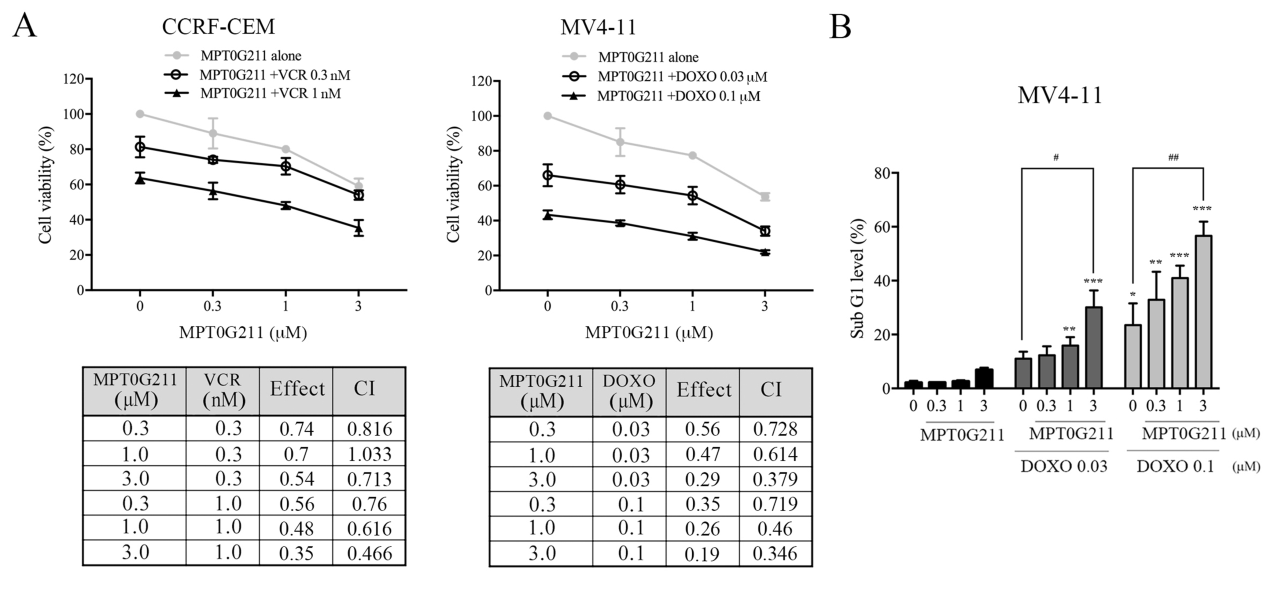


**Figure S1. The combination effect of MPT0G211 and chemotherapeutic agent on acute leukemia cell lines.**

(A) The viability of cells treated with MPT0G211 and different doses of doxorubicin (DOXO) or vincristine (VCR) for 48 h was tested on CCRF-CEM and MV4-11 cell lines. The synergistic effects of these drugs were evaluated using the combination index (CI), with values of >1.0, 1.0, and <1.0 indicating an antagonistic, additive, or synergistic interaction, respectively. (B) Cell cycle distribution was determined in cells treated with various concentrations of MPT0G211 and DOXO for 48 h on MV4-11 cells.
